# The Epidemiology of Vertigo, Dizziness, and Unsteadiness and Its Links to Co-Morbidities

**DOI:** 10.3389/fneur.2013.00029

**Published:** 2013-03-22

**Authors:** Alexandre Bisdorff, Gilles Bosser, René Gueguen, Philippe Perrin

**Affiliations:** ^1^EA DevAH – Development, Adaption and Disadvantage, Cardio-Respiratory Regulations and Motor Control, Faculty of Medicine and UFR STAPS, Université de LorraineNancy, France; ^2^Department of Neurology, Centre Hospitalier Emile MayrischEsch-sur-Alzette, Luxembourg; ^3^Department of Cardiac Rehabilitation, Regional Institute for Rehabilitation, University HospitalVandoeuvre-lès-Nancy, France; ^4^Centre for Preventive MedicineVandoeuvre-les-Nancy, France; ^5^Department of Oto-Rhino-Laryngology, University HospitalVandoeuvre-lès-Nancy, France

**Keywords:** epidemiology, vertigo, dizziness, migraine, agoraphobia, motion sickness, anxiety, vaso-vagal

## Abstract

Vertigo, dizziness, and unsteadiness (VDU) are common symptoms traditionally considered to result from different kinds of vestibular and non-vestibular dysfunctions. The epidemiology of each symptom and how they relate to each other and to migraine, agoraphobia, motion sickness susceptibility (MSS), vaso-vagal episodes (VVE), and *anxiety-depression* was the object of this population-based study in north-eastern France. A self-administered questionnaire was returned by 2987 adults (age span 18–86 years, 1471 women). The 1-year prevalence for vertigo was 48.3%, for unsteadiness 39.1%, and for dizziness 35.6%. The three symptoms were correlated with each other, occurred mostly (69.4%) in various combinations rather than in isolation, less than once per month, and 90% of episodes lasted ≤2 min. The three symptoms were similar in terms of female predominance, temporary profile of the episodes, and their link to falls and nausea. Symptom episodes of >1 h increase the risk of falls. VDU are much more common than the known prevalence of vestibular disorders. The number of drugs taken increase VDU even when controlling for age. Each VDU symptom was correlated with each co-morbidity in Chi-squared tests. The data suggest that the three symptoms are more likely to represent a spectrum resulting from a range of similar – rather than from different, unrelated – mechanisms or disorders. Logistic regressions controlling for each vestibular symptom showed that vertigo correlated with each co-morbidity but dizziness and unsteadiness did not, suggesting that vertigo is certainly not a more specific symptom than the other two. A logistic regression using a composite score of VDU, controlling for each co-morbidity showed a correlation of VDU to migraine and VVE but not to MSS and not to agoraphobia in men, only in women.

## Introduction

Vertigo and dizziness rank among the most common reasons for consultation and referral to specialist care (Sloane, 1989; Nakashima et al., 1996; Moulin et al., 2003). A methodological problem affecting studies on vertigo and dizziness is the uneven use of these terms by the public and professionals (Blakley and Goebel, 2001).

An approach to the patient based on the quality of four mutually exclusive symptoms which would predict the underlying cause has been propagated since the 1970s (Drachman and Hart, 1972) and is still widely taught: (i) vertigo is due to vestibular causes, (ii) presyncope to cardio-vascular causes, (iii) disequilibrium to neurological causes, and (iv) non-specific dizziness to either psychiatric or metabolic causes. Recent studies have shown that this approach is flawed and potentially dangerous, particularly in the emergency setting (Stanton et al., 2007; Newman-Toker et al., 2008).

The international vestibular community has started to classify vestibular disorders. A first step was to propose definitions of symptoms, which are distinctive, non-overlapping and not mutually exclusive entities, and without any hypothesis regarding etiology or mechanism (Bisdorff et al., 2009).

The vestibular system works at a subconscious level and serves many purposes related to oculo-motor control, balance regulation, and perception of self-motion (Massion and Woollacott, 1996). It comprises the labyrinthine part of the inner ear and its connections in the brain stem and cerebellum. However, it also has widespread cortical connections and the particular feature of being multimodal (integrating vestibular, proprioceptive, and visual inputs) even at the very basic level of the vestibular nuclei (Cullen, 2012). Dysfunctions can lead to a wide range of symptoms: from basic perceptual symptoms like vertigo, dizziness, visual and balance symptoms to problems of emotion, memory, and self-perception (Borel et al., 2008).

Vertigo and dizziness are also frequently associated with other common diseases and conditions, such as migraine (Neuhauser et al., 2001), motion sickness (Dai et al., 2007), faints (Newman-Toker et al., 2008), and anxiety (Staab and Ruckenstein, 2003; Eckhardt-Henn et al., 2008).

This study sets out to examine the prevalence of vertigo, dizziness, and unsteadiness (VDU) in an adult population. To avoid the problem of the uneven use of terms, it uses purely phenomenological definitions for each. It also makes it possible to establish the co-occurrence of the three symptoms and whether they relate differently to markers of severity for balance disorders like falls and to certain co-morbidities of vestibular disorders.

## Materials and Methods

In the *Centre de Médecine Préventive* of Nancy Vandoeuvre (Lorraine region, north-eastern France) the nursing staff distributed and collected questionnaires from 3035 adults ≥18 years who had volunteered to take part in this study. From adolescence through old age, French people are regularly invited to local centers of preventive medicine to have a free health check-up. The data were recorded anonymously and the study followed the rules of the local ethical committee [*comité de protection des personnes* (CPP)].

The study used a self-administered questionnaire, which included some basic demographic data and a medical part. The questions on VDU were taken from the vestibular part of the Vertigo Symptom Scale (VSS) (Yardley et al., 1992), which describes to the participant the various symptoms in a way that can easily be understood, leaves little room for interpretation, and does not use the technical terms vertigo or dizziness. The descriptions provided are close to the newly proposed definitions (Bisdorff et al., 2009) and can therefore be easily transposed into the corresponding medical term:

- “a feeling that things are spinning or moving around”: vertigo,
- “a feeling of being light-headed, ‘swimmy’ or giddy”: dizziness,
- “feeling unsteady, about to lose balance”: unsteadiness.

For each item participants ticked the duration and frequency of episodes over the last year. Two items refer to severity: balance-related falls and “incapacity to stand or walk without support” ( = severe unsteadiness). The developer of the VSS defined two composite scores:

- vertigo short for VDU symptoms lasting for up to 1 h (VDUshort),
- vertigo acute for VDU of >1 h plus severe unsteadiness, falls, and nausea (VDUacute).

For the composite scores a ≥4 cut-off was chosen so as to have a significant level of symptoms.

Further inclusions were the migraine identifier (Lipton et al., 2003), the Motion Sickness Susceptibility (MSS) Questionnaire (MSSQ) (Golding, 1998), and vaso-vagal episodes (VVE) (Bosser et al., 2006). Markers for agoraphobia and anxiety-depression (AD) were taken from the Marks fear questionnaire (Marks and Mathews, 1979), using the French translation (Cottraux et al., 1987). The maximum score was 40 each, with a cut-off of >10.

### Statistical methodology

To correct for differences between the sample and the general population of Lorraine, weights were defined for gender, age, and level of education. In each category the weights were the ratio of the proportion in the general population to the observed proportion in the sample. All statistical analyses and tests were performed with weighted data. The statistical package used was SPSS 16.0 for windows.

Bivariate associations were tested by applying Chi-squared tests. Multivariate logistic regression was used, with forward stepping, to explain binary variables from several quantitative and qualitative covariates. Odds-ratios (OR) estimates were derived from these logistic models.

## Results

Of the 3035 questionnaires returned 2987 (1471 women) were included, after rejecting those with too many missing or unusable data. The average age (*y*) was 45.6 for men and 46.2 for women with a similar distribution.

### Global prevalence of vestibular symptoms

**Figure 1:**
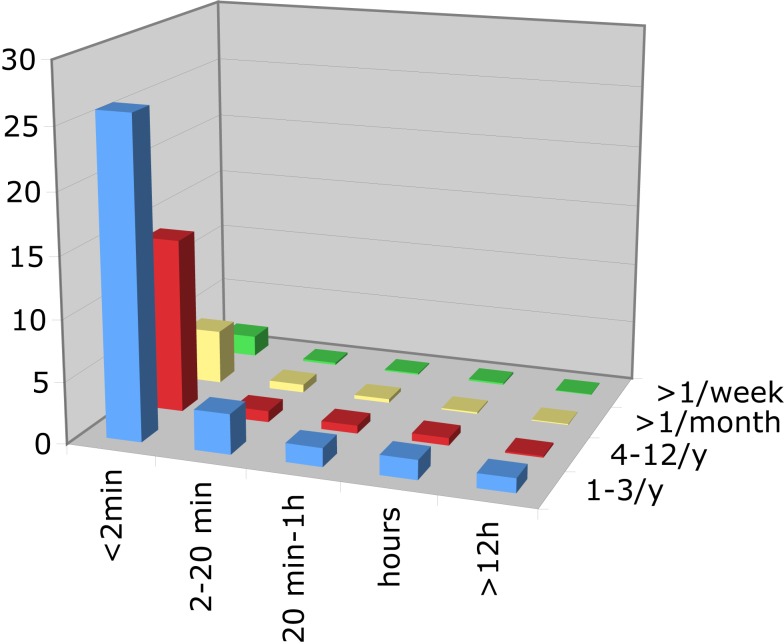
**The *z*-axis represents the prevalence in %, the *x*-axis the duration of vertigo-episodes, and the *y*-axis the frequency of vertigo-episodes**.

At least one VDU symptom had occurred at least once in the last 12 months in 59.2% of participants, vertigo (48.3%) was followed by unsteadiness (39.1%) and dizziness (35.6%). About 90% of each symptom episodes were of short (≤2 min) duration and occurred less than once per month (Table 1). The histogram according to frequency and duration for the three symptoms are similar (Figure 1 displays the example of vertigo).

**Table 1:** **One-year prevalence (%) of VDU symptoms according to duration and frequency**.

Frequency		<2 min	2–20 min	20 min–1 h	Hours	>12 h
1–3/year	Vertigo	25.98*	3.26*	1.52	1.58	1.17
	Dizziness	19.44	2.59	1.25	1.29	0.97
	Unsteadiness	22.71	2.20	1.35	1.19	1.03
4–12/year	Vertigo	14.21*	0.92	0.66	0.61	0.14
	Dizziness	10.18	1.32	0.44	0.23	0.22
	Unsteadiness	9.89	1.19	0.53	0.4	0.21
>1/month	Vertigo	4.42*	0.67	0.31	0.12	0.09
	Dizziness	2.41	0.77	0.25	0.14	0.08
	Unsteadiness	2.94	0.61	0.29	0.20	0.15
>1/week	Vertigo	1.71	0.18	0.10	0.14	0.06
	Dizziness	1.03	0.24	0.13	0.11	0.00
	Unsteadiness	1.28	0.25	0.10	0.04	0.05

Some participants had short as well as long lasting symptoms, therefore the sums for each symptom are higher than the global prevalence indicated in the text. (*Vertigo more prevalent *p* < 0.01).

Most participants (69.4%) with symptoms experienced more than one type in various combinations (Table 2). The three symptoms were significantly correlated, as were those with short and long durations.

**Table 2:** **The prevalence of combinations of VDU and balance-related fallers, severe unsteadiness, and nausea**.

	*N* = 1514 % Male	*N* = 1468 % Female	*N* = 2982 % Total	Fallers (%/Group)	Severe unsteadiness (%/Group)	Nausea
No sympt	49.6	31.7	40.8	1.2	1.1	20.5
Vert only	9	11.8	10.4	2.0	2.9*	45.3*
Dizz only	3.5	2.6	3.1	0.9	1.1	46.0*
Vert + dizz	5.6	7.6	6.6	3.7	4.7*	51.3*
Unstead only	4.6	4.7	4.6	4.4	5.7*	48.0*
Unstead + vert	8	9.3	8.6	11.4*	11.9*	52.1*
Unstead + dizz	3.6	2.8	3.2	14.1*	17.3*	65.5*
Vert + dizz + unstead	16.1	29.6	22.7	16.4*	22.3*	68.1*

Vert, vertigo; dizz, dizziness; unstead, unsteadiness. (*p < 0.01).

The risk for falls and severe unsteadiness was significantly increased if symptoms came in combinations rather than in isolation, particularly if the combination included unsteadiness.

The risk of nausea was similarly increased for each VDU symptom – and still further in case of symptom combinations.

Balance-related falls and severe unsteadiness were about three times more frequent if the symptoms lasted >1 h rather than ≤1 h, and this was true for all three symptoms.

### Gender and age effect

In women the symptoms were more frequent than in men in most age groups except for ≥70 years. The prevalence was similar across the various age groups except that in women VDUacute was lower for age group 40–49 years (Table 3).

**Table 3:** **Percentages per age group and gender comparison for VDUshort, VDUacute, falls, and severe unsteadiness**.

Age groups (years)	VDUshort		VDUacute		Fallers		Severe unsteadiness	
	M/F (%)	*p*	M/F (%)	*p*	M/F (%)	*p*	M/F (%)	*p*
19–29	21/31	<0.01	4/12	<0.01	5/11	<0.05	5/13	<0.02
30–39	12/33	<0.01	4/12	<0.01	6/8	ns	6/10	<0.01
40–49	15/29	<0.01	3/5	ns	4/7	ns	3/11	<0.01
50–59	14/34	<0.01	2/12	<0.01	6/8	ns	6/12	<0.01
60–69	14/21	<0.01	4/8	ns	4/8	<0.01	6/9	ns
≥70	17/17	ns	7/12	ns	3/8	ns	5/7	ns

### Medication and VDU

Leaving aside contraception, 55.4% took no medication and 4.4% more than six drugs. The number of drugs increased with age. The prevalence of VDUshort and VDUacute increased significantly with the number of drugs, independently of age and gender (Table 4). The risk for VDUshort and VDUacute decreased with age when controlling for drug intake. Balance-related falls increased strongly with five or more drugs but were also mildly increased with exactly one drug. Each VDU symptom was significantly correlated to drug intake in Chi-squared tests, but in a logistic regression controlling age and gender and for each VDU symptom, unsteadiness was the best explanatory factor.

**Table 4:** **Number of drugs, age, and VDU**.

*N*	Age groups									
	436	538	587	558	632	220	2971			
Number of drugs	18–29 years (%)	30–39 years (%)	40–49 years (%)	50–59 years %	60–69 years %	≥70 years %	Total %	VDUshort % (OR)	VDUacute % (OR)	Falls % (OR)
0	85.6	74.3	72.1	46.8	25.8	11.8	55.4	18.5 (1)	4 (1)	5.1 (1)
1	8.9	16.4	14.8	22.0	16.9	11.8	14.7	24.6 (1.58)	9.5 (2.78)	9.8 (2.2)
2	3.4	4.3	5.3	18.1	13.1	26.4	10.3	23.8 (1.85)	6.3 (2.13)	5.3 (1.3) ns
3	1.4	3.3	1.9	8.1	16.5	15.0	7.3	15.9 (1.18) ns	5.6 (2.02)	6.6 (1.7) ns
4	0.2	1.1	1.9	5.7	11.1	8.2	4.6	28.1 (2.72)	7.9 (3.43)	3.6 (1.0) ns
5	0	0.2	2.0	3.0	7.1	9.1	3.2	27.7 (2.74)	15.6 (7.80)	13.2 (4.1)
≥6	0.5	0.4	1.9	3.2	9.5	17.7	4.4	40.2 (4.95)	19.8 (9.35)	12.3 (3.8)
VDUshort (OR)	1	0.77 ns	0.68	0.65	0.33	0.27				
VDUacute (OR)	1	0.84 ns	0.35	0.45	0.26	0.38				

*The two columns on the right display the % and OR for VDUshort, VDUacute, and balance-related falls adjusted for age, gender, and number of drugs, taking zero drug as reference = 1. The two lower rows the OR for age groups adjusted for number of drugs, gender and age, taking group 18–29 as reference = 1; ns = not significant*.

### VDU and co-morbidities

The global prevalence of migraine was 17.90% with the typical female predominance and decrease over age 50 years, which is similar to European figures (Stovner and Andree, 2010).

The average agoraphobia score was 4.1 which is comparable with data from the literature (Mizes and Crawford, 1986). Prevalence of moderate or severe MSS in men was 3.9% and in women 11.2%, in a study using the same tool (Bosser et al., 2006) in a population of students <30 years, the prevalence were 6.0% for men and 15.7% for women, which is also comparable considering that MSS tends to decrease with age.

Each VDU symptom was highly correlated with every co-morbidity in Chi-squared tests (*p* < 0.001). Table 5 sets out the results of the logistic regression analysis testing the correlation of the co-morbidities with each VDU symptom, controlling for age, gender, and the other VDU symptoms. In this model migraine is correlated with each VDU symptom, VVE and AD with vertigo and dizziness, MSS and agoraphobia only with vertigo.

**Table 5:** **Logistic regression for each co-morbidity controlling for gender, age, and each VDU symptom**.

	Vertigo	Dizziness	Unsteadiness
Migraine	1.11 (1.01–1.22)	1.36 (1.21–1.52)	1.14 (1.03–1.26)
MSS	1.18 (1.09–1.26)	ns	ns
VVE	1.31 (1.20–1.43)	1.18 (1.08–1.29)	ns
Agoraphobia	1.32 (1.22–1.41)	ns	ns
AD	1.22 (1.16–1.34)	1.23 (1.12–1.36)	ns

*MSS, motion sickness susceptibility; VVE, vaso-vagal episodes; AD, anxiety-depression. (OR 95% CI)*.

Anxiety-depression and female gender significantly increased the risk for VDUshort, VDUacute, and all co-morbidities except AD for VVE. A separate multivariate regression analysis was therefore conducted in men and women with and without AD (Table 6).

**Table 6:** **VDU and co-morbidities controlled for gender and anxiety-depression**.

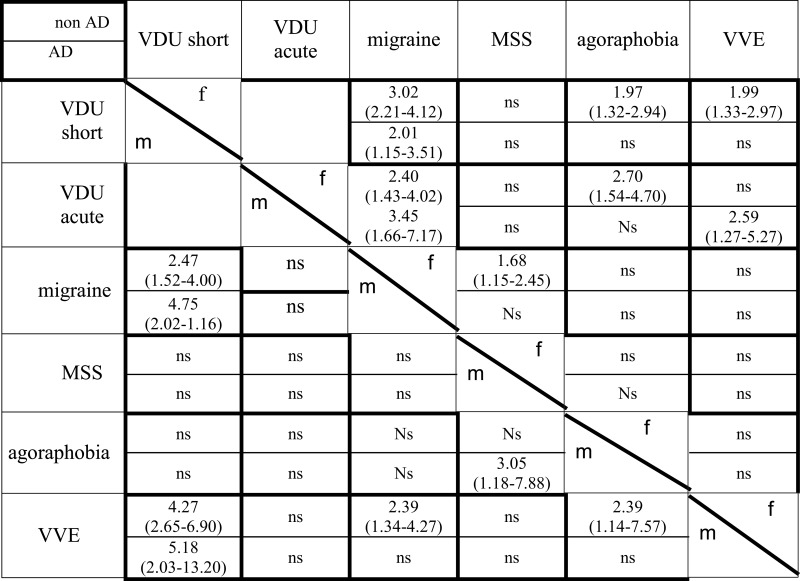

*The upper part of each cell shows data for non-AD, the lower for AD participants. Data for men under diagonal line and for women above. AD, anxiety-depression; VVE, vaso-vagal episodes; MSS, motion sickness susceptibility; VDUshort, composite score of VDU symptoms ≤ 1 h; VDUacute, composite score of VDU symptoms > 1 h*.

Migraine increased the risk for VDUshort in men and women and for VDUacute in women, independently of AD.

Vaso-vagal episodes increased the risk for VDUshort in men with and without AD, in women without AD, and for VDUacute in AD women.

In the global analysis the highest OR for MSS were found for female gender 2.53 (CI 1.92–3.34), followed by AD (OR 2.04; CI 1.51–2.76) and migraine (OR 1.48; CI 1.10–1.98). When controlling for AD and gender, migraine was not significant in men but still in non-AD women, MSS did not increase the risk for VDUshort or VDUacute.

In the global analysis the highest risks for agoraphobia were AD (OR 3.48; CI 2.63–4.61) and female gender OR 2.36 (CI 1.78–3.11). When the data were controlled for gender and AD, agoraphobia was found to increase the risk for VDUshort, VDUacute in non-AD women. In men the only factor was the combination of AD and MSS.

## Discussion

Vertigo, dizziness, and unsteadiness have a high and roughly even prevalence throughout adult life. The three symptoms are correlated with each other, usually appear in short episodes and in various combinations rather than in isolation (Tables 1 and 2), have a similar link to nausea, severe unsteadiness, and balance-related falls, all have a female predominance (Table 3) and all are correlated to each co-morbidity evaluated in this study.

This challenges the traditional view that vertigo is a more specific symptom resulting from dysfunction of the inner ear or its immediate connections in the brain stem and cerebellum (Perrin et al., 2011), whereas dizziness might result from non-acute vestibular disorders but also from a much wider range of causes like metabolic, cardio-vascular, or psychiatric disorders (Baloh, 1996). The present data suggest that the three symptoms are more likely to represent a spectrum resulting from a range of similar – rather than from different, unrelated – mechanisms or disorders. The lack of specificity of vestibular symptoms has also been pointed out in clinical studies in acute vestibular patients (Stanton et al., 2007). This population-based study seems to be in line with this concept, but cannot directly support it as the mechanisms in relatively healthy volunteers and seriously ill patients are likely to be of a different nature.

Occasional and short lasting and isolated symptoms are very common in the population of all ages and might not mean anything serious. On the other hand combinations of symptoms and those lasting for >1 h were correlated with markers of severity like falls.

Previous epidemiological studies found a similar symptom prevalence (Hannaford et al., 2005; Neuhauser et al., 2005) but did not make such a clear distinction between VDU. The study by Neuhauser et al. (2005) found that vertigo/dizziness was about six times – and severe symptoms about three times – more prevalent than a narrower definition of vertigo resulting from vestibular disorders. VDUacute, which reflects symptoms of >1 h duration had a prevalence similar to “vestibular vertigo” as defined in that study.

This points to a big gap between the prevalence of VDU symptoms and that of vestibular diseases. Indeed, the sum of the 1-year prevalence of the most common vestibular disorders – benign paroxysmal positional vertigo 1.6% (von Brevern et al., 2007), vestibular migraine 0.89% (Neuhauser et al., 2006), Menière’s disease 3.5–513/100000 (Alexander and Harris, 2010), and vestibular neuritis annual incidence of 3.5/100000 (Strupp and Brandt, 2009) – is well below 5%. It is not clear how this gap is to be explained, but broadly two, mutually non-exclusive, explanations should be considered. First, the spectrum of vestibular disorders might be wider than captured by the present diagnostic criteria. As the definitions are usually driven by needs of diagnostic certainty, they tend to give more weight to specificity. The consequence is that milder forms of disease presentation may not meet the diagnostic criteria. The second factor could be that VDU also arise from a range non-vestibular conditions, and this explicitly also applies to vertigo. The study gives some suggestions of which factors might play a role.

Drugs might be an important factor (Table 4). There is a clear increase for VDU and falls with the number of drugs taken, and drug consumption goes up with age. It is not obvious how to interpret the finding that consumption of exactly one drug also mildly increased VDU. Each VDU symptom correlated with drug intake but unsteadiness is the symptom best explained by drugs in the logistic regression analysis controlling for vertigo, dizziness, and age. It is well established that drugs increase the risk of falls in the elderly (Woolcott et al., 2009), and vertigo/dizziness is a common side-effect of many drugs like anti-hypertensives and sedatives.

A surprising finding is that when controlling for drug intake, VDU decreased with age.

This suggests that drugs or the diseases for which they are prescribed explain a part of VDU, particularly in the elderly and those elderly people who do not need to take any or only a few drugs are very healthy and fall less. On the other hand, in young adults who take little or no medication, the VDU prevalence is similar to older adults, who usually take many drugs. In young adults, factors like migraine and VVE seem to be more important for explaining VDU.

All co-morbidities examined in this study were correlated with each VDU symptom, which suggests they arise from common factors, but each symptom might also have a specific component. In logistic models controlling for each vestibular symptom, age and gender some differences appeared (Table 5). Vertigo was correlated with each co-morbidity, whereas dizziness and unsteadiness were not. This would mean that vertigo is even less, but definitely not more, specific than dizziness or unsteadiness. VVE was correlated with vertigo and dizziness but not unsteadiness. There have traditionally been doubts as to whether orthostatic hypotension could induce spinning vertigo and in an epidemiological survey on orthostatic hypotension (Radtke et al., 2011) vertigo was explicitly excluded. However, recent data have clearly established that a spinning sensation can be observed in primary cardio-vascular conditions (Newman-Toker et al., 2008), and our data suggest that VVE could explain part of the prevalence of dizziness as well as vertigo, particularly among young people and women, where VVE were more prominent.

In this model agoraphobia was only correlated with vertigo and AD with vertigo and dizziness. One might have expected from the data from behavioral and emotional complications of vestibular disorders that dizziness would come out more prominently here rather than vertigo (Ruckenstein and Staab, 2009; Staab, 2012). Migraine was the only co-morbidity correlated to all three VDU symptoms which suggests that from migraine the whole range of vestibular symptoms could arise as opposed to the more narrow definition of vestibular migraine proposed recently (Lempert et al., 2012).

The co-morbidities examined in this study are known to be interconnected. Migraine has a link to MSS (Marcus et al., 2005), VVE to MSS (Bosser et al., 2006), agoraphobia to anxiety (Kessler et al., 2006), and all to vertigo. Therefore a logistic model was done to see how they relate when controlling for each other and VDU before and after and separating genders and AD from non-AD because VDU and co-morbidities were higher in women and in AD, except AD in VVE (Table 6).

Migraine has a robust link to VDUshort in men and women independently of AD and other co-morbidities, in women also to VDUacute. This finding supports the importance of migraine for VDU.

Vaso-vagal episode has a link to VDUshort in non-AD-men and women when controlling for the other co-morbidities and suggest it to be an important factor for short lasting vestibular symptoms, particularly in the young and middle aged and is in line with a recent German study (Radtke et al., 2011).

From the global results the main risk factors for MSS were AD and female gender. When controlling for these factors MSS was not linked to VDU and not to migraine in men and only weakly in women. Since migraine and anxiety are more common in women, this could explain why studies in migraine patients showed high rates of MSS (Marcus et al., 2005) but migraine as such might not be the main factor.

In the global analysis the strongest explanatory factors for agoraphobia were AD and female gender. When controlling for gender and AD, VDU increased the risk of agoraphobia in women but not in men. In AD-men MSS increased the risk of agoraphobia. The interface of anxiety and vestibular disease is a complex topic (Balaban et al., 2011), from our data the difference between women and men is not explained by migraine and that AD is more important than VDU.

The prevalence of VDU was found to be high across all age groups but tended to decrease in the age group >70. In other studies there was usually an increase with age, particularly in studies focusing on elderly populations of 65–90 year-olds (Jönsson et al., 2004; Stevens et al., 2008; Gassmann and Rupprecht, 2009). In a study encompassing the adult population up to 70 years (Hannaford et al., 2005) there was a mild increase with age in men, but not in women. The findings in the >70 years in our study are probably explained by a recruitment bias. To get to the *Centre de Médecine Préventive*, persons need to come by their own means. People who are seriously ill or disabled people or who live in institutions would normally not come to this kind of health check because they would be followed up in a different kind of setting. This selection bias is likely to be more important with increasing age. The >70 years group was the smallest in our study, and our weighting correction could probably not control for the selection bias for healthier elderly adults.

## Conflict of Interest Statement

The authors declare that the research was conducted in the absence of any commercial or financial relationships that could be construed as a potential conflict of interest.
